# Bailey’s Maneuver With an I-gel®: A Smooth Extubation in an At-Risk Cardiac Patient

**DOI:** 10.7759/cureus.70504

**Published:** 2024-09-30

**Authors:** Utkarshini Kedia, Sankalp Goel

**Affiliations:** 1 Anesthesiology, Dr. D. Y. Patil Medical College, Hospital and Research Center, Dr. D. Y. Patil Vidyapeeth (Deemed to Be University), Pune, IND; 2 Plastic Surgery, Dr. D. Y. Patil Medical College, Hospital and Research Center, Dr. D. Y. Patil Vidyapeeth (Deemed to Be University), Pune, IND

**Keywords:** bailey's maneuver, deep extubation, hemodynamic stability, high-risk cardiac, i-gel®, supraglottic airway device

## Abstract

Each extubation requires careful monitoring due to the potential for complications such as increased airway reflexes and iatrogenic injuries. Here, we describe a case involving a Whipple’s procedure for carcinoma of the head of the pancreas in a 71-year-old high-risk cardiac patient. The patient successfully underwent extubation using Bailey’s maneuver, in conjunction with an I-gel® supraglottic airway device (SAD), without any exaggeration of the hemodynamic response. This technique allowed for a controlled and smooth extubation, ensuring cardiovascular stability in this high-risk case.

## Introduction

Difficult intubations are well-known scenarios encountered by anesthetists, intensivists, and surgeons. However, difficult extubations are less commonly recognized or understood. Extubation is defined as the purposeful removal of the tracheal tube, transitioning from an established airway to a normal, natural airway [1]. It is crucial that each extubation is carefully monitored, as complications can arise due to increased airway reflexes, iatrogenic injuries, and other factors. The All India Difficult Airway Association (AIDAA) provides stepwise guidelines for extubation in various clinical situations, such as normal airways with awake or deep extubation, difficult mask ventilation, and suspected airway edema, among others [2].

One of the methods described in the AIDAA guidelines is Bailey’s maneuver for extubation, which involves the exchange of an endotracheal tube with a laryngeal mask airway (LMA) at the end of surgery while the patient is still in a deep plane of anesthesia. This approach helps minimize the risk of adverse airway events during the transition from mechanical ventilation to spontaneous breathing.

In this report, we describe the successful use of Bailey’s maneuver in the extubation of a high-risk cardiac patient undergoing a Whipple’s procedure for carcinoma of the head of the pancreas. This method was selected due to the patient's underlying cardiac condition and the need to reduce the risk of a pressor response during extubation. The careful application of this technique allowed for a smooth transition and uneventful recovery in this high-risk patient.

## Case presentation

A 71-year-old male presented with fever, vomiting, and abdominal pain. Upon further investigation, he was diagnosed with carcinoma of the head of the pancreas and was scheduled for a Whipple's procedure under general anesthesia.

**Figure 1:**
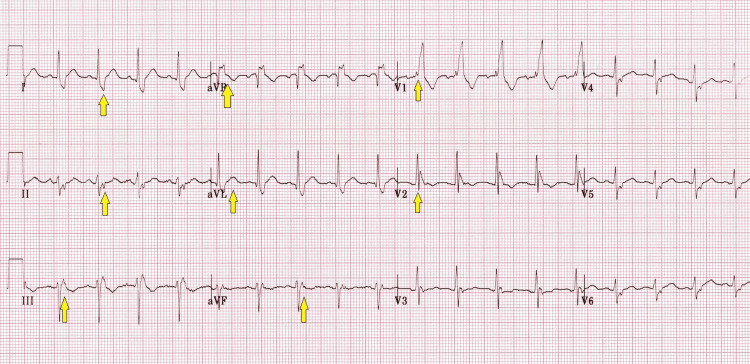
Patient's abnormal pre-operative ECG. Yellow colored arrows: suspicious changes ECG, electrocardiogram

The patient had a history of hypertension, managed for the past three months with amlodipine 5 mg daily. His electrocardiogram (ECG) revealed ST segment changes and a hemiblock pattern (Figure 1). As part of the preoperative cardiac evaluation, a 2D echocardiogram and a dobutamine stress examination (DSE) were conducted. The DSE was positive for induced ischemia and hypotensive response. There was no regional wall motion abnormality, no change in the ejection fraction, and no symptoms of induced cardiac failure in any of the stages of the test. The patient had an uneventful recovery after the DSE. Hence, the cardiologist gave fitness for surgery due to high cardiac risk, with precaution to smooth induction, maintenance, and extubation without inducing a stress response. As Whipple's procedure is a time-sensitive procedure, delay in intervention may worsen the condition of the patient for which any cardiac intervention was not deemed necessary by the cardiologist. Clinical examination of the respiratory and circulatory systems was unremarkable, and the patient remained hemodynamically stable. Systemic examination was within normal limits. His hemoglobin level was 11.2 g/dL, and his platelet count was 2.98 lakh/mm³.

On airway examination, the mouth opening of the patient was 3 cm with Mallampati grading II. The patient did not have any dental abnormalities. The laryngeal handshake maneuver could be performed adequately. The thyromental and hyomental distances were normal with adequate temporal-mandibular joint function, and neck extension was not restricted.

High risk was explained to the patient and their relatives, and consent for anesthesia was taken. General anesthesia was planned with oral intubation by a channeled video-laryngoscope.

After taking the patient into the operation theatre, an 18G intravenous cannula was secured and the patient was premedicated with an injection of glycopyrrolate 0.2 mg IV, injection of ranitidine 45 mg IV, and injection of ondansetron 4 mg IV. Maintenance fluid of Ringer's lactate was started through the intravenous cannula. The patient was pre-oxygenated with 100% oxygen at 15 liters per minute flow for three minutes at CPAP of 10 cm of H_2_O. The patient was induced with 100 µg fentanyl, 100 mg propofol, and 40 mg rocuronium IV. Once apneic, the patient was ventilated for three minutes with PCV mode of the ventilator with 100% O_2_, as per institutional protocol. A channeled video-laryngoscope was introduced into the midline of the oral cavity with a preloaded, 7.0 mm cuffed endotracheal tube. The endotracheal tube was gradually passed under indirect vision and fixed. Anesthesia was maintained on oxygen, air, and desflurane. PCV mode was set with peak inspiratory pressure of 14 cm of H_2_O, frequency of 14 liters per minute, FiO_2_ of 60%, and PEEP at 6 cmH_2_0. A nasogastric tube was inserted, which was also requested by the surgeon for post-operative monitoring. Invasive blood pressure monitoring was accomplished with a left radial arterial catheter. A 7 French central line was inserted in the right internal jugular vein and an epidural catheter was inserted at the lower thoracic level (T11-T12) for analgesia, which was to be continued post-operatively. Surgery was started and was uneventful. 

**Figure 2:**
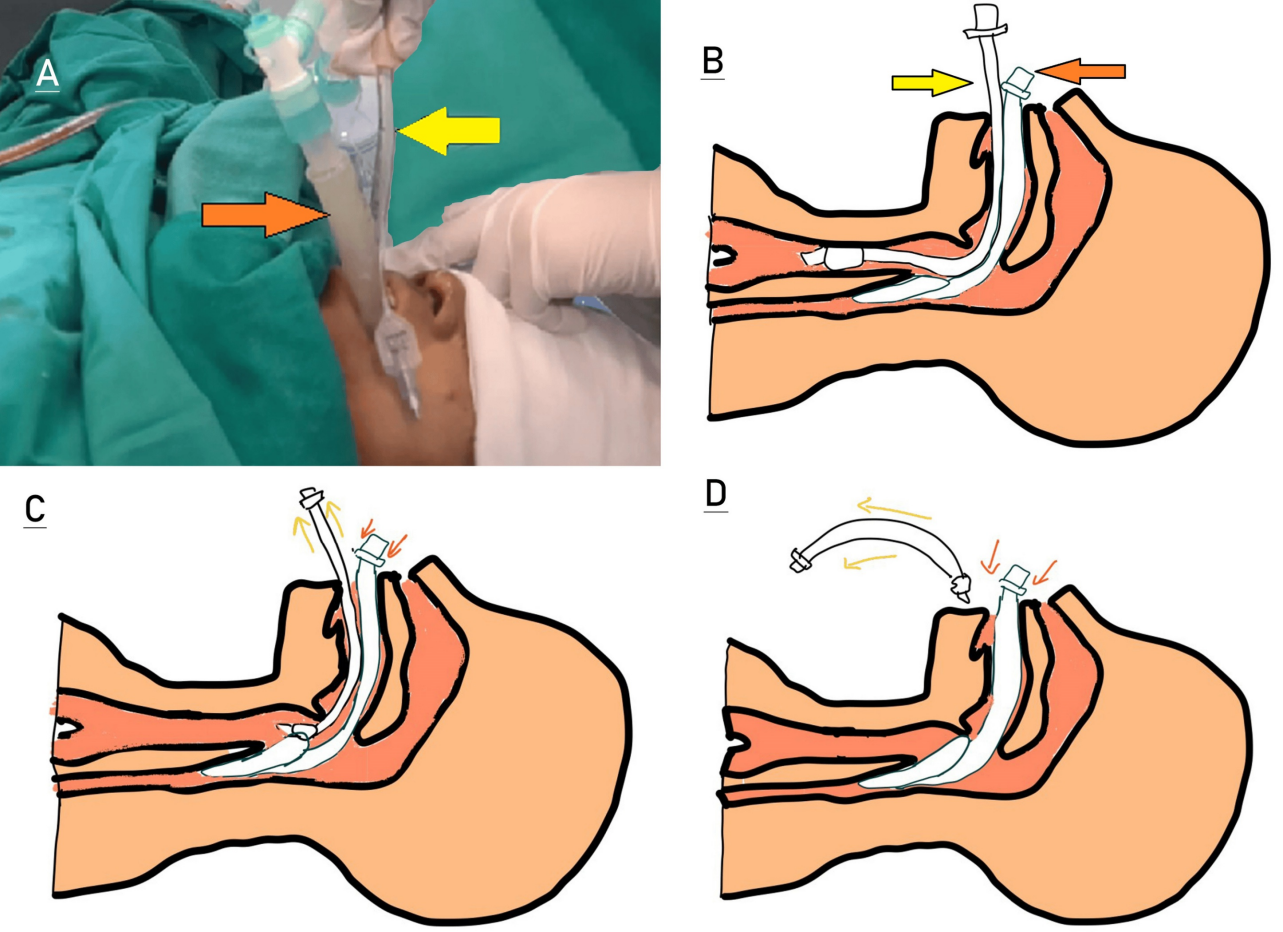
Bailey's maneuver with I-gel®. A) Clinical image with I-gel® (orange arrow) and endotracheal tube (yellow arrow) in situ. B) Diagrammatic representation with both in situ. C) While  I-gel® is in situ (orange arrows), the endotracheal tube is being pulled out (yellow arrows). D) I-gel® (orange arrows) in situ with extubation of endotracheal tube (yellow arrows). Orange arrow: I-gel® supraglottic airway device (SAD); yellow arrow: endotracheal tube. Planes B, C, and D are the author's own creations.

The risk of pressor response resulting in an increase in blood pressure and heart rate during normal extubation was high, which may have aggravated the underlying cardiac issues of the patient. In order to decrease bucking, coughing, and pressor response, the decision was made to proceed with Bailey’s maneuver for extubation. The oral cavity was thoroughly suctioned. An I-gel® no. 4 was introduced behind the endotracheal tube still in situ with its cuff inflated. The cuff of the endotracheal tube was deflated, the ventilator circuit was attached to I-gel® ensuring adequate ventilation through the space surrounding the tube, and the endotracheal tube was removed. Ventilation was continued with the in situ I-gel® after adjusting it to its favorable position as shown in Figure 2. The neuromuscular blockade was gradually reversed with injection of sugammadex 200 mg and the patient started taking spontaneous breaths. Desflurane was stopped when the patient had fully recovered from the effect of neuromuscular blockade. The I-gel® was removed when the patient was awake and following verbal commands. The patient was transferred to the Surgical Intensive Care Unit (SICU) and was kept under observation. The patient was hemodynamically stable and had an uneventful recovery.

## Discussion

Bailey’s maneuver was first introduced by Paul Bailey in 1995, emphasizing the safety of using an LMA to extubate patients under deep anesthesia [3]. This technique is particularly suitable for patients who have undergone surgeries where suppression of respiratory and cardiovascular reflexes is critical, such as ocular, intracranial, thoracic, and thyroid surgeries, or in cases where cardiovascular stability during extubation is essential [4].

In our case, the patient was a high-risk cardiac case, necessitating a smooth extubation without triggering cardiovascular reflexes. Tracheal extubation can provoke a pressor response, characterized by a 10-30% increase in both blood pressure and heart rate, lasting for five to 15 minutes. This was an unacceptable risk for our patient. The AIDAA guidelines suggest that replacing the endotracheal tube with a SAD during deep anesthesia is an effective method to attenuate the pressor response. Extubation in this state can help prevent adverse hemodynamic and respiratory responses such as hypertension, tachycardia, dysrhythmias, coughing, laryngospasm, myocardial ischemia, and increases in intracranial and intraocular pressures. However, deep extubation carries inherent risks of upper airway obstruction and aspiration. To minimize these risks, the use of a SAD, ideally a second-generation SAD, is recommended [2].

In this case, we employed the I-gel®, a second-generation SAD, which features a non-inflatable cuff that fits snugly into the airway. The unique design of the I-gel® prevents pressure necrosis due to its snug fit without requiring inflation. The smooth, contiguous undersurface of the I-gel® allows it to slide easily along the posterior pharynx into the hypopharynx, facilitating insertion. Additionally, the I-gel® acts as a bite block, which was originally used externally in Bailey’s 1995 description of the maneuver [3]. The device also includes an integrated epiglottic rest, which prevents downfolding of the epiglottis, further reducing the risk of airway obstruction [5].

In this patient, the I-gel® was inserted posterior to the endotracheal tube. After deflating the endotracheal tube cuff, the tube was removed, leaving the I-gel® in place. Its design enabled a straightforward insertion and provided adequate airway management without complications. Moreover, the availability of the I-gel® in most clinical settings makes it a practical choice for Bailey’s maneuver. Its wide airway passage also allows for the reinsertion of an endotracheal tube, if needed.

In this case, the use of Bailey’s maneuver with the I-gel® facilitated a smooth and uneventful extubation, achieving the desired outcome in this high-risk cardiac patient.

## Conclusions

Extubation under deep anesthesia can effectively prevent undesirable hemodynamic and respiratory responses, such as hypertension, tachycardia, dysrhythmias, coughing, laryngospasm, myocardial ischemia, and increases in intracranial and intraocular pressures. However, this technique also poses risks, including upper airway obstruction and aspiration. To mitigate these risks, the use of a SAD as a bridging solution is recommended. This approach is particularly advantageous in neurosurgical, thoracic, ophthalmic, and ENT surgeries, as well as in patients with ischemic heart disease, like the case presented here.

In this patient, Bailey’s maneuver was employed in combination with an I-gel® SAD, which facilitated a smooth and controlled extubation. This technique provided an effective solution for managing the patient's airway while minimizing the risk of triggering cardiovascular reflexes, crucial in a high-risk cardiac patient. The successful use of Bailey’s maneuver with the I-gel® allowed for a safe and uneventful extubation.
